# Hypoglycemic Activity and the Potential Mechanism of the Flavonoid Rich Extract from *Sophora tonkinensis* Gagnep. in KK-Ay Mice

**DOI:** 10.3389/fphar.2016.00288

**Published:** 2016-09-05

**Authors:** Mi Huang, Shihao Deng, Qianqian Han, Ping Zhao, Qi Zhou, Sijian Zheng, Xinhua Ma, Chan Xu, Jing Yang, Xinzhou Yang

**Affiliations:** ^1^School of Pharmaceutical Sciences, South-Central University for NationalitiesWuhan, China; ^2^State Key Laboratory of Drug Research, Shanghai Institute of Materia Medica, Chinese Academy of SciencesShanghai, China; ^3^College of Biological Engineering, Tianjin University of Science and TechnologyTianjin, China

**Keywords:** hypoglycemic agents, *Sophora tonkinensis* Gagnep., KK-Ay mice, GLUT4, p-AMPK

## Abstract

This study investigated the active principles, hypoglycemic activity and potential mechanisms of the flavonoid rich extract from *Sophora tonkinensis* Gagnep. (ST-EtOAc) in KK-Ay diabetic mice. An off-line semipreparative liquid chromatography-nuclear magnetic resonance (LC-NMR) and liquid chromatography-ultraviolet-electrospray ionization mass spectrometry (LC-UV–ESIMS) protocol was performed to determine 13 flavonoids from ST-EtOAc. ST-EtOAc administrated orally to the KK-Ay mice significantly increased their sensibility to insulin, reduced fasting blood-glucose levels and blood lipid indexes such as triglyceride and cholesterol. Moreover, ST-EtOAc exhibited a strong effect of stimulation on glucose transporter 4 (GLUT4) translocation by 2.7-fold in L6 cells. However, the selective AMP-activated protein kinase (AMPK) inhibitor compound C can completely inhibit the activation of the AMPK pathway and prevent the GLUT4 translocation caused by ST-EtOAc. *In vivo*, phosphorylation of the AMPK expression in the liver and skeletal muscle was measured. The results showed phosphorylation of the AMPK had been improved and GLUT4 expression had been also enhanced. In this paper, we conclude that, ST-EtOAc seems to have potential beneficial effects on the treatment of type 2 diabetes mellitus with the probable mechanism of stimulating GLUT4 translocation modulated by the AMPK pathway.

## Introduction

Type 2 diabetes mellitus (T2DM), a metabolic disorder of the endocrine system characterized by abnormal glucose and lipid metabolism, is caused by insulin resistance and relative insulin deficiency (Shulman, 2000). Because insulin resistance is the main metabolic abnormality of T2DM, there has been considerable interest in insulin-sensitizing agents for the treatment of this disease (Moller, 2001; Ju et al., 2014). One of the most appealing targets for drug development is the insulin-responsive glucose transporter 4 (GLUT4), which is vital to glucose homeostasis (Bryant et al., 2002). Increasing evidence suggests that enhanced translocation of GLUT4 can improve insulin resistance of T2DM. Therefore, this protein may lead to the discovery of the next generation of anti-diabetic drugs (Zhang et al., 2007; Tsuchiya et al., 2015).

Many traditional Chinese medicines (TCMs), which contain many active compounds targeting GLUT4 proteins, have revealed beneficial hypoglycemic effects *in vitro* and *in vivo*, such as *Coptis chinensis* (Turner et al., 2008), *Momordica charantia* (Tan et al., 2008), and *Gardenia jasminoides* (Zhang et al., 2006), etc. In order to identify potential hypoglycemic agents to fight T2DM, we developed a L6 cell-based GLUT4 translocation system with co-expressing recombinant GLUT4 and insulin regulation of aminopeptidase (IRAP) using a confocal imaging technique to screen the extracts or fractions from natural products (Wang et al., 2014; Yang X.Z. et al., 2014; Yang et al., 2015). During the screening of a TCMs extract library (800 biotas) on GLUT4 translocation, we have found that a flavonoid rich extract of *Sophora tonkinensis* Gagnep. (ST-EtOAc) had a potentially beneficial effect on GLUT4 translocation.

*Sophora tonkinensis* Gagnep. belongs to the leguminous family, and has been widely used as a traditional medicine in China (Tan et al., 2009). Clinically, it is mainly used to treat acute and chronic pharyngitis, tonsillitis, and chronic hepatitis (Sheng et al., 2010). However, the hypoglycemic effect of *S. tonkinensis* Gagnep. has not been reported until now. The aim of this investigation was to determine the active principles, and evaluate the potential hypoglycemic effects of ST-EtOAc *in vitro* and *in vivo*, and therefore elucidate its probable hypoglycemic mechanism.

## Materials and Methods

### Instruments and Reagents

Liquid chromatography-photo-diode array-electrospray ionization mass spectrometry (LC-PDA-ESIMS) data were recorded on a Waters ACQUITY SQD MS system (Waters, Milford, MA, USA) connected to a Waters 1525 high performance liquid chromatography (HPLC) with a 2998 Photodiode Array Detector (Waters, Milford, MA, USA) and a Waters Sunfire^TM^ C18 column (5 μm, 4.6 × 150 mm) (Waters, Ireland). The nuclear magnetic resonance (NMR) spectra were recorded in dimethyl sulfoxide (DMSO)-d6 on an spectrometer (AVANCE III 500 MHz) equipped with 1.4 mm heavy wall Micro NMR tubes (NORELL, Landisville, NJ, USA). All the other analytical-reagent grade chemicals were used with no further purification, and HPLC-grade solvents were obtained from Merck Chemical Company (Darmstadt, Germany). The NMR (high-Resolution solution: MeOH-d4 or DMSO-d6) spectra were kept a record in on an AVANCE III 500 MHz spectrometer equipped with Micro NMR tubes (1.4 mm). Typically, ^1^H spectra and ^1^H-^1^H COSY spectra were separated obtained at the scan range of 32–128 and 4–32. The heteronuclear multiple bond correlation (HMBC) and the heteronuclear single quantum coherence (HSQC) spectra were recorded at the scan range of 16–96.

### LC–PDA–ESIMS Method

Analysis was performed on a C_18_ column (Waters Sunfire, 5 μm, 4.6 × 150 mm). 0.1% formic acid added in Water (A) and acetonitrile (B) was used as mobile phases. The gradient elution was used for analysis and the program was as follows: 0–20 min, 10–100% B; 20–24 min, 100% B. The flow rate of the analysis was 1.0 mL/min. UV-Vis spectra were obtained with the wavelength range of 200–500 nm with 10 scans per second. The splitted eluent was at a ratio of 1:5 before the mass spectrometer. Both positive and negative ion modes were used for ESIMS recording. The capillary voltage was 4000 V, the capillary exit voltage was 140.0 V, and the skimmer voltage was 40 V. The nebulizer gas pressure was set to 40 psi, the dry gas flow to 10.0 L/min and the dry temperature to 320°C. Mass range was set from 120 to 1500 *m/z*. Data acquisition and processing were achieved with MassLynx^TM^ 4.0 software (Waters, Milford, MA, USA).

### Plant Material and Preparation of ST-EtOAc

The roots of *S. tonkinensis* Gagnep. were collected from Jingxi county, Guangxi Zhuang Autonomous Region, China in June 2013. And it was identified by Professor Jingquan Yuan (Guangxi Medicinal Botanical Garden, Nanning, China). The plant voucher specimen was preserved as No. SC0060 and deposited in College of Pharmacy, South-Central University for Nationalities. Air-dried roots of *S. tonkinensis* Gagnep. (500 g) were smashed and then extracted sequentially at room temperature with *n*-hexane (4 × 2.0 L, 3 h each), followed by ethyl acetate (4 × 2.0 L, 3 h each) and methanol (4 × 2.0 L, 3 h each). The solvents were vacuum evaporated to yield *n*-hexane extract (4.9 g), ethyl acetate extract (ST-EtOAc, 36.8 g), and methanol extract (58.7 g), respectively.

### Chemical Characterization

Separation of ST-EtOAc was carried by the procedures as follows: 0.5 g of ST-EtOAc was dissolved in 2.0 mL of DMSO so that a concentration of ST-EtOAc was 250 mg/mL, and then the solution was filtered. With the same solvent system to that of LC–PDA–ESIMS, the optimized gradient program used as follows: 0–25 min, 15–100% B; 25–30 min, 100% B. The flow rate was set at 5.0 mL/min and the 200 μL injected volume was setup. Ten injections were carried out, and 13 peak-based fractions were collected manually. And combined the corresponding fractions. Final purification were performed as follows: peaks 1–6, 10, 12, and 13 were filtered by a Sephadex LH-20 column (400 mm × 10 mm, MeOH containing 0.1% formic acid) to yield pure compounds **1** (2.9 mg), **2** (2.1 mg), **3** (3.8 mg), **4** (3.5 mg), **5** (1.9 mg), **6** (23.3 mg), **10** (37.6 mg), **12** (3.2 mg), and **13** (7.1 mg). Peak 7 was purified by preparative thin layer chromatography (TLC) (hexane:acetone:formic acid—100:10:0.5) to yield compound **7** (1.2 mg). Peak 8 was purified by preparative TLC (hexane:acetone:formic acid—100:10:0.4) to give compound **8** (1.8 mg). Peak 9 was purified by preparative TLC (hexane:acetone:formic acid—100:8:0.4) to give compound **9** (2.8 mg). Peak 11 was purified by preparative TLC (hexane:acetone:formic acid—100:8:0.4) to give compound **11** (1.4 mg). Compounds **1–13** with amounts 0.8–3.0 mg were dissolved in DMSO-d_6_ or MeOH-d_4_ for NMR tests on a spectrometer (AVANCE III 500 MHz) which was equipped with 1.4 mm heavy wall Micro NMR tubes.

### Plasmid and Cell Line Construction

pIRAP-mOrange cDNAs, presented by Professor Xu Tao (Institute of Biophysics, Chinese Academy of Sciences), were inserted into the pQCXIP plasmid. The retrovirus was prepared by transfecting pQCXIP-IRAP-mOrange, vesicular stomatitis virus glycoprotein (VSVG), and PHIT60 with a ratio of 2:1:1 by lipofectamine 2000 into PLATELET cells, collecting the cultural supernatant after 48 h, and concentrating the viruses by supercentrifuge (50,000 *g*, 30 min). L6 cells at the exponential growth phase were infected with fresh prepared viruses. The polybrene (Millipore, 8 μg/mL) was used to facilitate the infection efficiency. The red fluorescence cells were isolated by fluorescence activated cell sorter (FACS) and single cell was seeded into 96 well-plates. Finally the single clone, which had the highest increase in red fluorescence intensity following stimulation with insulin (100 nM), was selected.

### IRAP Translocation Assay

GLUT4 has mainly been recruited to the plasma membrane (PM) throughout to the GLUTs storage vesicles (GSV). Three main proteins stored in GSV are GLUT4, IRAP, and Sortilin (Shi and Kandror, 2005). Many researches reported that IRAP and GLUT4 displayed a strong colocalization (Rubin and Bogan, 2009; Kumar et al., 2010). Thus, detecting the IRAP can indirectly reflect the situation of GLUT4. The methodology validation could be found in S1 in the Supplementary Material.

L6 cells which stably express IRAP-mOrange were cultured in minimum essential media (MEM)-α supplemented with 10% fetal bovine serum (FBS) and 1% antibiotics (100 U/mL penicillin and 100 μg/mL streptomycin) at 37 C in 5% CO_2_. L6 IRAP-mOrange was seeded in 48 well plates, and incubated until 100% confluence and then starved in serum-free MEM-α for 2 h. Afterward, L6 cells were treated with samples and other agents. Compound C {6-[4-(2-piperidin-1-ylethoxy)phenyl]-3-pyridin-4-ylpyrazolo[1,5-a] pyrimidine, commonly used as an inhibitor of AMP-activated protein kinase (AMPK)} and Wortmannin {(1S,6bR,9aS,11R,11bR)-11-(acetyloxy)-1, 6b, 7, 8, 9a, 10,11,11b-octahydro-1-(methoxy-methyl)-9a,11b-dimethyl-(3H-Furo[4,3,2-de]indeno[4,5-h]-2-benzopyran-3,6,9-trione, commonly used as a phosphatidylinositol 3-kinase (PI3K) inhibitor)} was used to investigate the mechanism. The cells photos were taken with a laser-scanning confocal microscope LSM 510 (Carl Zeiss, Jena, Germany) to supervise the IRAP-mOrange translocation. And the images were captured with 555 nm excitation laser every 10 s in first 5 min and then every 5 min in later 25 min.

### Determination of Glucose Uptake in L6 Myocytes

MEM-α, FBS, and antibiotics (100 U/mL penicillin and 100 μg/mL streptomycin) were purchased from Hyclone, USA. L6 Cells were maintained in MEM-α and used for experiment described previously (Al-Maharik and Botting, 2008; Li et al., 2008; Lee et al., 2015). A cell-based 2-[*N*-(7-nitrobenz-2-oxa-1,3-diaxol-4-yl)amino]-2-deoxyglucose (2-NBDG) Glucose Uptake Assay Kit (Cayman Chemical, USA) was used to measure the effects of ST-EtOAc in promoting the glucose uptake on L6 cells. L6 cells were seeded in a 96-well plate with 1 × 10^4^–5 × 10^4^ cells/well in 100 μL MEM-α medium. After 12 h incubation, the myotubes were treated with different concentration of ST-EtOAc, 10 μM compound C (an AMPK inhibitor) and 5-aminoimidazole-4-carboxamide 1-β-D-ribofuranoside (AICAR) (an AMPK agonist) or normal control in 100 μL (150 μg/mL 2-NBDG) glucose-free MEM-α medium. After 24 h incubation, the glucose uptake of L6 cells was measured as the method described by the Cayman assay kit.

### Preparation of Protein in L6 Myotubes and Western Blotting

L6 cells (5 × 10^5^ cells) were subcultured into 60 mm dishes and cultured for 7 days to form myotubes in 3 mL of MEM-α with 2% FBS. After incubation, the L6 myotubes were treated with AICAR (1 mM), compound C (40 μM), ST-EtOAc, or vehicle (0.1% DMSO) for 60 min. The total protein samples were extracted from L6 myotubes, and western blotting for AMPK and phospho-AMPK was conducted as described previously (Yang J. et al., 2014; Yang et al., 2015). The antibodies specific for AMPKα (No. 2532), p-AMPKα (Thr172; No. 4188) were purchased from Cell Signaling Technology (Danvers, USA).

### Animals and Treatments

All experimental procedures were reviewed and approved by the Animal Ethical Committee of the Institute of Health and Epidemic Prevention (Wuhan, China; the protocol number 2015-SCUEC-AEC-0026), and animal care was conducted in accordance with institutional guidelines. The KK-Ay mice (*n* = 65, 8 weeks old, male) and C57BL/6J mice (*n* = 10, 8 weeks old, male) were purchased from the Beijing HFK Bioscience Co, Ltd (SCXK 2009-0015). All mice were individually housed in laminar flow cabinets under specific pathogen-free conditions. The KK-Ay mice were given a high-fat diet purchased from Medicience Co., Ltd., Yangzhou, China. The composition of the diet was as follows: protein, 225 g/kg; fat, 200 g/kg; carbohydrate substances, 450 g/kg; cholesterol, 12.5 g/kg; sodium cholate, 5 g/kg; energy, 4500 kcal/kg. After consecutive 4 weeks feeding, the average weight of KK-Ay reached 43 g and the fasted blood glucose levels of the mice were tested. The fasted blood glucose levels ≥11.1 mmol/L were classified as T2DM, and there were 50 mice up to the standard. These T2DM mice were divided randomly into five groups: vehicle group (group I, saline treatment, *n* = 10), ST-EtOAc treatment (group II–IV, dose of 60, 120, 240 mg/kg/day, *n* = 10/group), and Metformin treatment group (group V, 200 mg/kg/day, *n* = 10), the C57BL/6J mice (*n* = 10) were given standard laboratory diet (Beijing HFK Bioscience Co., Ltd) and as a normal control with saline. KK-Ay mice were feed with high-fat diets and C57BL/6J mice were feed with standard laboratory diet in the whole experimental period. All the groups were intragastric administration one time every day for four consecutive weeks.

The body weights were weekly recorded. Fasted blood glucose levels were measured weekly by an OneTouch blood glucose meter (Lifescan Inc., Wayne, USA). An oral glucose tolerance test (OGTT) was performed in mice after 12 h fasting at the 26th day of treatment. Blood glucose taken from the tail tip at 0, 30, 60, and 120 min after glucose administration was measured using a blood glucose meter (OneTouch Ultra^®^, Lifescan Inc., Wayne, USA). The glucose load was 2.0 g/kg orally. At the end of the experiment, the mice were fasted for 12 h. Blood samples were collected by retro-orbital sinus puncture using capillary tubes under diethyl ether anesthesia. Then, the blood samples were centrifuging at 3000 rpm for continues 15 min so that the serum of each sample was obtained. At the same time, the mice were euthanized by cervical dislocation. And the livers, skeletal muscles, and other tissues were harvested. Parts of livers and pancreas were immediately stored in liquid nitrogen tank, and the rest parts were stored in 10% neutral buffered formalin to be fixed.

### Liver and Pancreas Histological Analysis

Livers and pancreas fixed in formalin were embedded in paraffin, and then they were cut into 5 μm-thick sections and stained with standard hematoxylin–eosin (HE). The stained tissues were prepared for histopathological examinations, and pathological changes of the lesion and its vicinity in liver and pancreas were observed and photographed through an optical microscope photographed (200×). The average islet size (in μm^2^), was determined with software (program Stereo Investigator, Williston, VT, USA).

### Biochemical Analyses of Serum and Tissues

The serum levels of insulin, total cholesterol (TC), triglycerides (TG), low density lipoprotein cholesterol (LDL-C), and high density lipoprotein cholesterol (HDL-C) were determined by automatic biochemical analyzer (Hitachi 7180+ISE, Tokyo, Japan). Free fatty acid (FFA) was determined by corresponding assay kits (Jiancheng Bioengineering Institute, Nanjing, China). The tissue TC, TG, and FFA in mice were determined according to the method described previously (Lee et al., 2006).

### Tissue Extracts and Western Blotting

The total protein was extracted from skeletal muscle and liver according to the methods described previously (Yang et al., 2015). And the supernatant protein concentration was determined with bicinchoninic acid (BCA) assay kit (Abgent, San Diego, USA). An equivalent amount of samples were mounted on 10% sodium dodecyl sulfate (SDS)-polyacrylamide gel electrophoresis. The protein transferred electrophoretically to polyvinylidene fluoride membrane (Pall Corporation, Washington, USA) and incubated overnight at 4°C with antibodies specific for GLUT4 (No. 2213), AMPKα (No. 2532), p-AMPKα (Thr172; No. 4188; Cell Signaling Technology, Danvers, USA). The membranes were washed three times (10 min/wash) in tris buffered saline tween (TBST). Immune complexes were incubated with a peroxidase-conjugated antibody for 1 h. The blots were incubated in enhanced chemiluminescence kits (Amersham-Pharmacia, Piscataway, NJ, USA). And the immunoreactive signals were imaged and quantified with the Gel Image system (Aplegen Inc., Pleasanton, USA).

### Acute Toxicity Study

Kung Ming (KM) mice were derived in 1944 from a pair of Swiss mice that had been introduced from Hoﬄine Institution of Hindustan into Kunming of China (Shang et al., 2009). Kunming mice are the most commonly used outbred mouse line in China. This type of mice shows strong disease resistance and adaptability, high breeding coefficient and survival rate (Shang et al., 2009). So, KM mice have been widely utilized in pharmacological, toxicological, medicinal, and biological research and testing.

KM mice [No. SCXK (E) 2008-0005] weighing between 18 and 22 g were served as acute toxicity test. The animals were housed environmentally controlled conditions at 25°C, 60% relative humidity, where 12 h dark–light cycles were maintained with food and water. In the main study, the drug (ST-EtOAc dissolved in saline) was administered orally given to 10 male mice and 10 female mice at doses of a single dose of (6500 mg/kg/day). KM mice were observed for clinical symptoms for 15 days. At the end of the experiments, all the animals were euthanized followed and gross pathological examinations were undertaken.

### Statistical Analysis

Data was shown as means ± standard error of the mean. One-way analysis of variance (ANOVA) was used for multiple group comparisons by Tukey’s *post hoc* test using GraphPad Prism 5.0 software package. *P*-values <0.05 were considered significant.

## Results

### Chemical Characterization of ST-EtOAc

**FIGURE 1:**
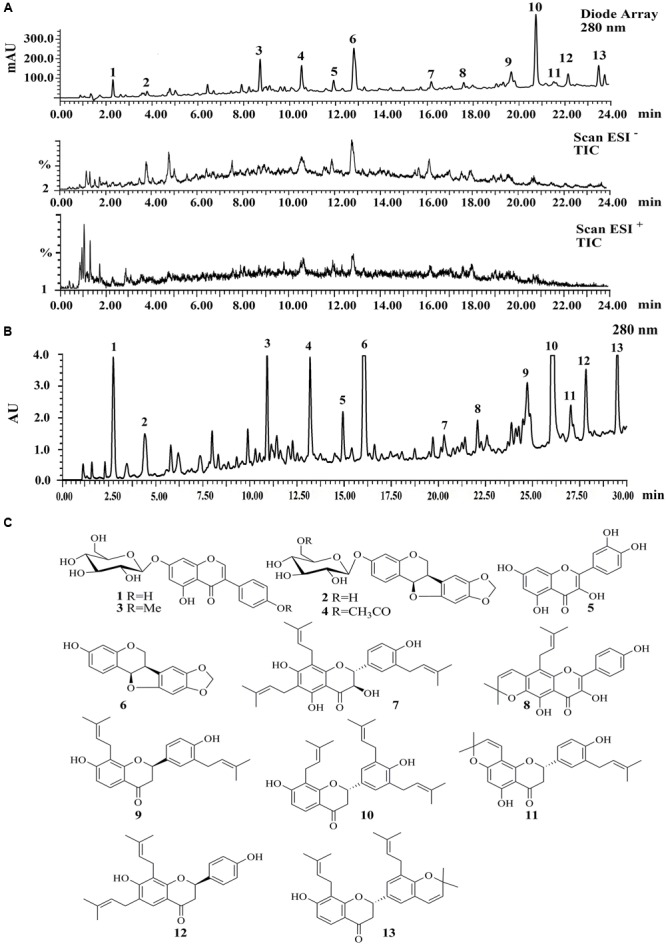
**(A)** The liquid chromatography-photo-diode array-electrospray ionization mass spectrometry (LC-PDA-ESIMS) analysis of ST-EtOAc is shown at 280 nm with peak labeling corresponding to compounds **1–13**. **(B)** The optimized semipreparative high performance liquid chromatography (HPLC) separation of the ST-EtOAc (50 mg in 200 μL dimethyl sulfoxide (DMSO)) is shown at 280 nm with peaks 1–13 collected for microprobe nuclear magnetic resonance (NMR) and further purified if required. **(C)** Structures of flavonoids from ST-EtOAc.

A LC–PDA–ESIMS experiment was carried out for chemical profiling of ST-EtOAc based on the standard operation procedure shown in Section “LC–PDA–ESIMS Method” (**Figure 1A**). The semipreparative HPLC chromatogram was used for large scale preparative isolation of target compounds with the optimized gradient conditions (**Figure 1B**). Thirteen peaks were collected and continue to further purified with Sephadex LH-20 column and preparative TLC to acquire 13 compounds for further 1D and 2D NMR spectroscopic analysis. The two major principles were determined as maackiain (**6**; Lee et al., 2015) and sophoranone (**10**; Li et al., 2008). The remaining compounds (**Figure 1C**) were identified as genistin (**1**; Al-Maharik and Botting, 2008), trifolirhizin (**2**; Yang X.Z. et al., 2014), ononin (**3**; Yang X.Z. et al., 2014), trifolirhizin 6′-monoacetate (**4**; Yang et al., 2014), quercetin (**5**; Yi et al., 2002), lespeflorin B4 (**7**; Mori-Hongo et al., 2009), dehydrolupinifolinol (**8**; Sutthivaiyakit et al., 2009), glabrol (**9**; Cho et al., 2012), euchrenone a2 (**11**; Mizuno et al., 1988), 6,8-diprenylkaempferol (**12**; Meragelman et al., 2001), and sophoranochromene (**13**; Li et al., 2008) by comparison with their mass spectrum (MS), UV, and NMR spectra with published reference data. Spectroscopic data of compounds 1–13 could be found in S2 in Supplementary Material.

### Effects of ST-EtOAc on GLUT4 Translocation, Glucose Uptake, AMPK Phosphorylation in L6 Myotubes

**FIGURE 2:**
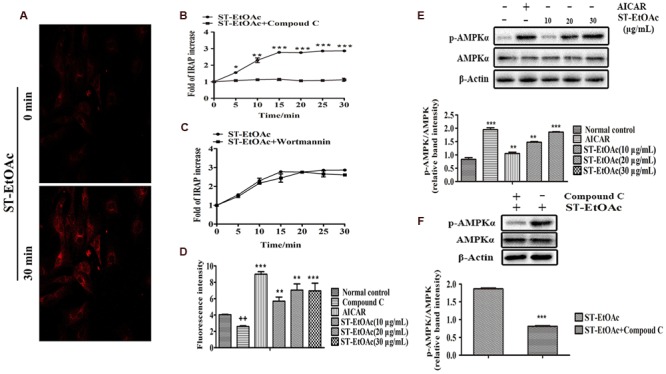
**ST-EtOAc stimulated IRAP tracking in L6 cell. (A)** L6 cells were infected with pIRAP-mOrange in order to detect externalized IRAP by confocal microscopy. Confocal images in L6 cells incubated in the absence (basal) or presence of ST-EtOAc for 30 min. **(B,C)** Data represent the fold increase in fluorescence induced by ST-EtOAc with inhibitors between 0 and 30 min. ^∗∗∗^*P* ≤ 0.001, ^∗∗^*P* ≤ 0.01, ^∗^*P* ≤ 0.05 compared to ST-EtOAc+Compound C group. **(D)** The effects of ST-EtOAc on stimulation of glucose uptake in L6 cells. ^++^*P* ≤ 0.01, compared to Normal control group. ^∗∗∗^*P* ≤ 0.001, ^∗∗^*P* ≤ 0.01, ^∗^*P* ≤ 0.05 compared to Normal control group. **(E)** The effect of ST-EtOAc on AMPK phosphorylation in L6 cells. **(F)** Compound C inhibits the effects of ST-EtOAc on AMPK phosphorylation. ^∗∗∗^*P* ≤ 0.001, compared to ST-EtOAc group.

As shown in **Figure 2A**, L6 cells stably expressing IRAP-mOrange were highly ST-EtOAc-responsive in terms of ST-EtOAc regulated IRAP translocation, and the effect on the translocation of IRAP was obviously enhanced by adding of ST-EtOAc. The results of mechanism study (**Figure 2B**) displayed that the translocation of IRAP to PM caused by ST-EtOAc stimulation was mainly inhibited by the prior adding of compound C (an inhibitor of AMPK). However, the addition of Wortmannin had no effect on the IRAP trafficking response (**Figure 2C**), suggesting that ST-EtOAc enhanced GLUT4 translocation by specifically targeting AMPK pathway. In the later study, the effect of ST-EtOAc stimulating glucose uptake in L6 cells was also tested. The promotion of glucose uptake was significantly inhibited by treatment with compound C. And comparing with the vehicle control, L6 cells were treated with different concentration of ST-EtOAc showing significantly effects on enhancing glucose uptake (**Figure 2D**). We performed western blotting assay using compound C and AICAR (an agonist of AMPK), to determine the regulatory mechanism by which ST-EtOAc induced the glucose uptake in L6 myotubes. As shown in **Figures 2E,F**, after adding of ST-EtOAc, the AMPK phosphorylation were increased in L6 cells compared with normal control and presented a dose-depending effect. However, the effects of AMPK phosphorylation caused by ST-EtOAc were repressed when adding compound C. So we can infer that glucose uptake enhancing in L6 cells by ST-EtOAc was modulated by activation of AMPK pathway.

### Effects of ST-EtOAc on Body Weight, Fasted Blood Glucose Levels, Oral Glucose Tolerance

**FIGURE 3:**
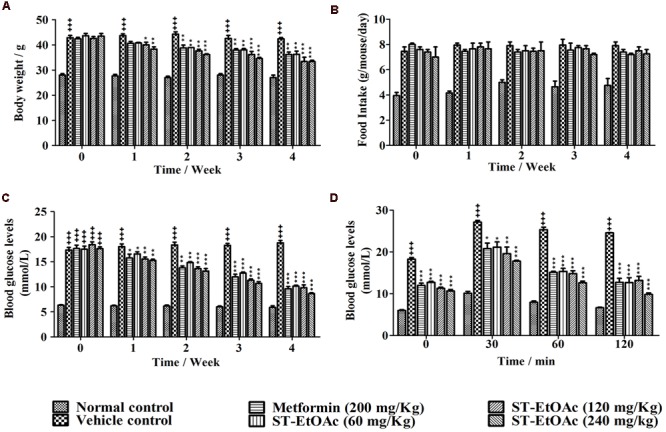
**Effects of ST-EtOAc on body weight, serum glucose level, OGTT, and food intake. (A)** The effects of ST-EtOAc on body weight. **(B)** The effects of ST-EtOAc on food intake. **(C)** The effects of ST-EtOAc on OGTT. **(D)** The effects of ST-EtOAc on fasted blood glucose level. ^+++^*P* ≤ 0.001, compared to normal control; ^∗∗∗^*P* ≤ 0.001, ^∗∗^*P* ≤ 0.01, ^∗^*P* ≤ 0.05, compared to T2DM mice treated with vehicle.

During the experiment, body weights of all groups of mice were monitored weekly (**Figure 3**). As shown in **Figure 3A**, the body weights of metformin and ST-EtOAc treated groups were reduced up in 4 weeks. Adversely, the vehicle control group had a little weight gain, compared to the initial weight. However, there were no significant differences in food intake between the vehicle group and ST-EtOAc groups during the 28 days treatment (**Figure 3B**). At the same time, we administered ST-EtOAc orally to KK-Ay mice to measure its glucose-lowering effect *in vivo*. As shown in **Figure 3C**, the treated mice had a steady and marked reduction in blood glucose level compared with vehicle group, whereas the control mice had no significant changes in the serum glucose level.

OGTT was also improved significantly, as evidenced by lower glucose levels at all time points after glucose loading. An OGTT was performed in mice at the 26th day of ST-EtOAc treatment. As shown in **Figure 3D**, an obvious improvement in OGTT was observed in metformin and ST-EtOAc groups. The data of Body Weight, Fasted Blood Glucose Levels and Oral Glucose Tolerance could be found in S3 in Supplementary Material.

### Effects of ST-EtOAc on Glucolipid Metabolism

**FIGURE 4:**
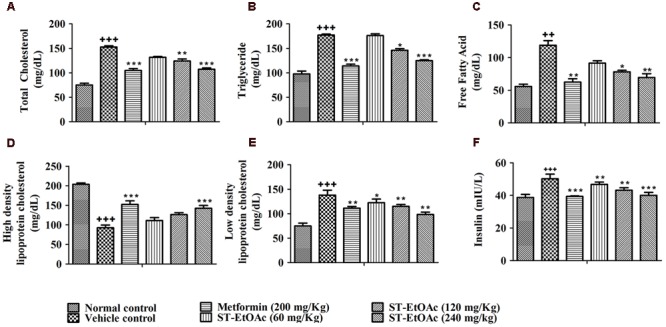
**(A–E)** Effects of ST-EtOAc on TC, TG, FFA, HDL-C, and LDL-C levels in the serum. **(F)** The effects of ST-EtOAc on serum insulin concentration. ^+++^*P* ≤ 0.001, ^++^*P* ≤ 0.01, compared to normal control; ^∗^*P* ≤ 0.01, ^∗∗^*P* ≤ 0.05, ^∗∗∗^*P* ≤ 0.001, compared to T2DM mice treated with vehicle.

**FIGURE 5:**
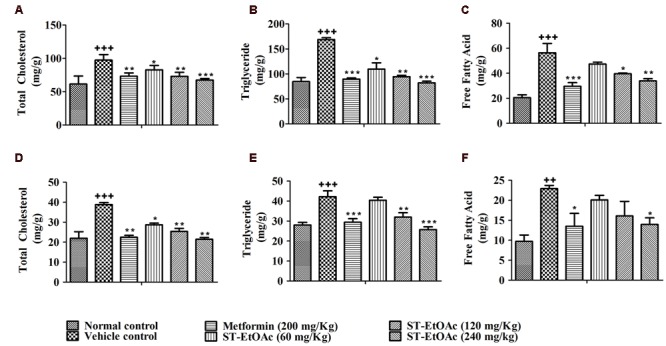
**Tissue lipid content after 4 weeks treatment.** TC **(A)**, TG **(B)**, and FFA **(C)** levels in mice liver tissue; TC **(D)**, TG **(E)**, and FFA **(F)** levels in mice skeletal muscle tissue. ^+++^*P* ≤ 0.001, ^++^*P* ≤ 0.01, compared to normal control; ^∗∗∗^*P* ≤ 0.001, ^∗∗^*P* ≤ 0.01, ^∗^*P* ≤ 0.05, compared to T2DM mice treated with vehicle.

We have examined the effects of ST-EtOAc on serum lipid parameters and adipose accumulation in liver and skeletal muscle in all mice. It was shown that ST-EtOAc significantly reduced the serum TC, TG, FFA, and LDL-C, and increased the serum HDL-C level in **Figures 4A–E**. Serum insulin was substantially reduced in the ST-EtOAc treated groups. The results showed that after treating, the insulin levels in treated groups were reduced significantly, illustrating ST-EtOAc can relieve the IR in KK-Ay mice (**Figure 4F**). TC, TG, FFA in skeletal muscle and liver had been reduced significantly throughout 4 weeks treatment by ST-EtOAc (**Figure 5**). The data of related index of Glucolipid Metabolism in KK-Ay mice could be found in S3 in Supplementary Material.

### Mouse Pancreas and Liver Histological Changes

**FIGURE 6:**
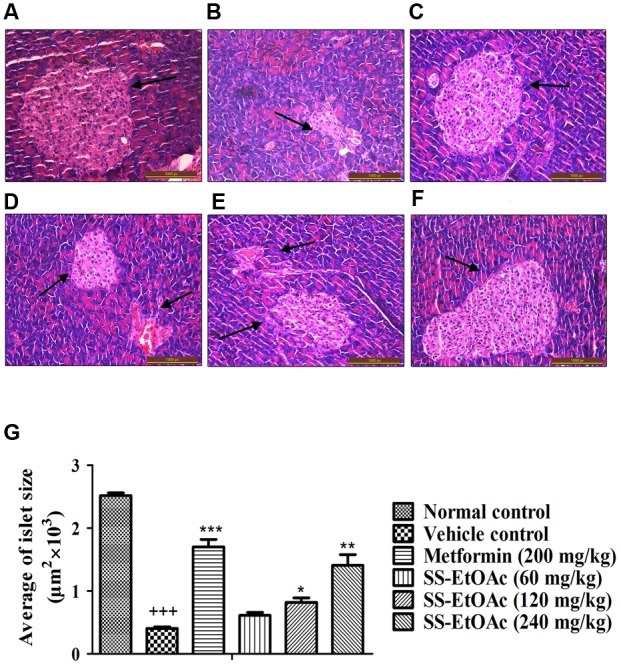
**Effectsof ST-EtOAc on morphological features of mice pancreas.** Optic microscopy: HE (×200). The arrows represent the islets of the mice pancreas. **(A)** Normal control. **(B)** KK-Ay mice treated with vehicle. **(C)** KK-Ay mice treated with metformin (200 mg/kg). **(D)** KK-Ay mice treated with ST-EtOAc (60 mg/kg). **(E)** KK-Ay mice treated with ST-EtOAc (120 mg/kg). **(F)** KK-Ay mice treated with ST-EtOAc (240 mg/kg). **(G)** Quantitative analysis of islet area. ^+++^*P* ≤ 0.001, compared to normal control; ^∗∗∗^*P* ≤ 0.001, ^∗∗^*P* ≤ 0.01, ^∗^*P* ≤ 0.05, compared to T2DM mice treated with vehicle.

**FIGURE 7:**
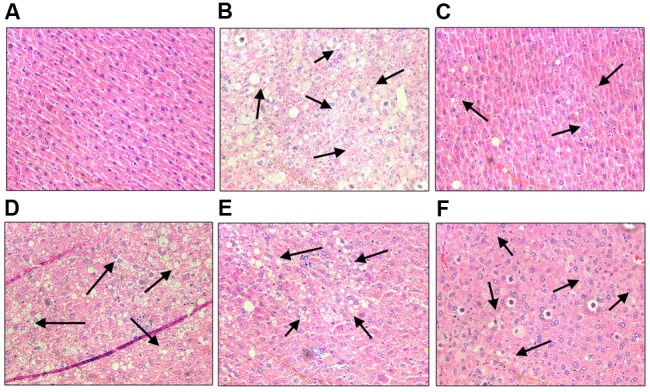
**Effects of ST-EtOAc on morphological features of mice livers.** Optic microscopy: HE (×200). The arrows represent the lipidosis in the mice livers. **(A)** Normal control. **(B)** KK-Ay mice treated with vehicle. **(C)** KK-Ay mice treated with metformin (200 mg/kg). **(D)** KK-Ay mice treated with ST-EtOAc (60 mg/kg). **(E)** KK-Ay mice treated with ST-EtOAc (120 mg/kg). **(F)** KK-Ay mice treated with ST-EtOAc (240 mg/kg).

In the study, we performed histological examinations to observe whether ST-EtOAc have the capacity of protecting pancreas in KK-Ay mice. The results showed that ST-EtOAc could alleviate the abnormity caused by diabetes in KK-Ay mice via a dose-dependent manner (**Figure 6**). In the vehicle group, the hepatic steatosis and empty lipid vacuoles appeared obviously in liver, however, after the treatment of ST-EtOAc for 4 weeks, the degree of hepatic steatosis and empty lipid vacuoles was reversed, especially in the 240 mg/kg ST-EtOAc group (**Figure 7**).

### Western Blot Analysis on Tissues

**FIGURE 8:**
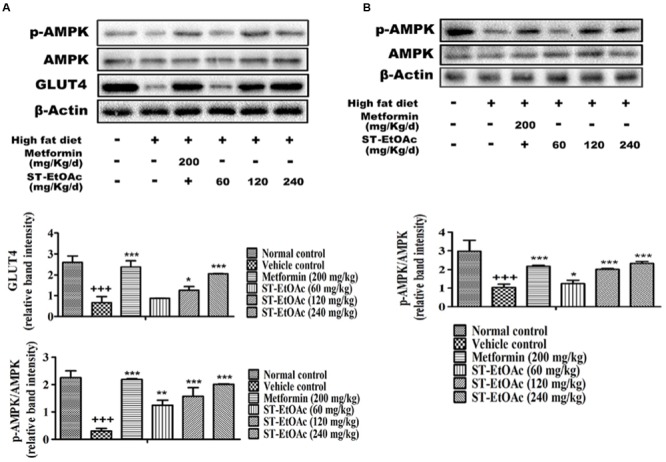
**(A)** Effects of ST-EtOAc on AMPK phosphorylation and GLUT4 expression in skeletal muscle. **(B)** Effects of ST-EtOAc on AMPK phosphorylation in liver. ^+++^*P* ≤ 0.001, compared to normal control; ^∗∗∗^*P* ≤ 0.001, ^∗∗^*P* ≤ 0.01, ^∗^*P* ≤ 0.05, compared to T2DM mice treated with vehicle.

In this study, we have demonstrated the possible AMPK-GLUT4 pathway of the effects of ST-EtOAc in L6 cells. *In vivo*, additionally, we examined the expression of p-AMPK and GLUT4 in the skeletal muscles. As expected, the results showed that the expression of GLUT4 and p-AMPK in skeletal muscles of T2DM mice had been improved. Especially, the 120 mg/kg/day group revealed the similar effects to the 240 mg/kg/day group in enhancing the phosphorylation of AMPK after 28 days treatment (**Figure 8A**).

At the same time, we have examined the phosphorylation of AMPK in liver by western blotting. As shown in **Figure 8B**, the levels of phosphorylated AMPK significantly were increased in the livers in KK-Ay mice treated with ST-EtOAc. These findings suggested that ST-EtOAc may active the AMPK pathway to attenuate lipogenesis in hepatocytes.

### Results of Acute Toxicity Study

During the acute toxicity study, 15 days observation period performed on ST-EtOAc treated KM mice have not produced mortality, abnormal clinical features, body weight disorders and food consumption, and toxicological changes in clinical biochemistry and hematology as well as other toxic signs (data has not been shown). The acute oral toxicity results showed that at the dose of 6500 mg/kg/day, ST-EtOAc concentrate is unlikely to be poisonous in KM mice.

## Discussion

Many TCMs have worked effectively served as adjuvants used to improve diabetic syndromes in combination with routine hypoglycemic drugs (Xie et al., 2011). Additionally, many TCMs, such as *Swertia macrosperma* (Wang et al., 2013), *M. charantia* (Tan et al., 2008), *G. jasminoides* (Zhang et al., 2006), and *C. chinensis* (Turner et al., 2008) etc., have been reported as having beneficial hypoglycemic effects *in vitro* and *in vivo*. Although many natural products have been used as hypoglycemic agents, many of them have not been studied deeply.

In order to look for potential hypoglycemic agents in natural products, we established a cell-based GLUT4 translocation assay using stable L6 cells expressing pIRAP-mOrange cDNAs. Our goal was to identify potential hypoglycemic plant extracts, fractions, and their isolated compounds by evaluating their effects on GLUT4 translocation. As we found, the plant extract ST-EtOAc displayed a strong effect on GLUT4 translocation by 2.7-fold in L6 cells. Based on the *in vitro* findings, we speculated that ST-EtOAc may have the hypoglycemic potency *in vivo*. And as we predicted, the *in vivo* data clearly validated the hypoglycemic activity of ST-EtOAc, including the ameliorative hyperglycemia and hyperinsulinemia in KK-Ay mice. The OGTT further indicated that hyperglycemia and hyperinsulinemia were considerably improved throughout ST-EtOAc treatment.

GLUT4 is the most important glucose transporter in skeletal muscle glucose metabolism (Huang and Czech, 2007), and it also plays an important role in insulin resistant, is the remarkable character in T2DM (Okamura et al., 2014). Promoting the GLUT4 translocation onto PM or expression in cells will relieve the T2DM. In this study, we found that ST-EtOAc had a strong stimulation on GLUT4 transportation onto PM. The expression of GLUT4 in skeletal muscle in KK-Ay mice treated with ST-EtOAc was elevated significantly, and presented a dose resistance according to the relationship. To evaluate the effects of ST-EtOAc on improving IR, indicators related to glycolipid metabolism in serum and tissues were examined. As the results shown in **Figures 3–5**, after 4 weeks of treatment with ST-EtOAc, body weight, and the blood glucose levels were significantly reduced in mice, and the OGTT showed that ST-EtOAc improved glucose tolerance. The serum insulin concentration had been dramatically reduced, and the OGTT showed the sensitivity of insulin had been improved. In the histopathological examination, ST-EtOAc also showed significant protection of the pancreas in KK-Ay mice. In addition, we observed a significant decline of TG, TC, and FFA levels in the serum of groups treated with ST-EtOAc. In another aspect, the level of HDL-C in the serum was in a certain degree improved in the KK-Ay mice treated with ST-EtOAc. Levels of TG, TC, and FFA in the liver and muscles were reduced significantly in T2DM mice treated with ST-EtOAc. Histopathological examination of mice livers showed that ST-EtOAc efficiently rescued liver steatosis associated with T2DM dose-dependently. These results showed that ST-EtOAc improved GLUT4 translocation and promoted expression level of GLUT4, thereby lowering blood glucose levels in mice and improving insulin resistance.

As previously reported, there are two signaling pathways leading to GLUT4 translocation to PM (Fullerton et al., 2012; Cazarolli et al., 2013), including the activation of PI3K and the activation of AMPK. In this study, when the L6 cells were incubated with compound C for 30 min before addition of ST-EtOAc, the translocation of GLUT4 to PM caused by ST-EtOAc stimulation was mainly inhibited by the addition of compound C (**Figure 2B**). However, the addition of Wortmannin had no effect on the IRAP trafficking response (**Figure 2C**). These results suggested that ST-EtOAc enhanced GLUT4 translocation by specifically targeting the AMPK pathway.

AMPK, an enzyme that regulates the body’s balance of energy, has been investigated as a potential therapeutic target for the treatment of T2DM (Fullerton et al., 2012). In glucose and lipid metabolism, the phosphorylation and activation of AMPK leads to GLUT4 translocation and eventually glucose uptake. It also acts primarily by directly affecting the activity of enzymes involved in carbohydrate, lipid, and protein biosyntheses and secondarily by long-term transcriptional control of key components (Rana et al., 2014). In our study, we detected the expression of p-AMPK in L6 cells and tissues using western blotting after administration of ST-EtOAc. As the data shown in **Figures 8A,B**, the content of p-AMPK in ST-EtOAc treated groups was significantly increased compared to the non-treated group. The result means that *in vitro*, ST-EtOAc enhanced the GLUT4 translocation through upregulating the level of p-AMPK in L6 cells. *In vivo*, AMPK in mouse skeletal muscle and liver were also detected, and western blotting data showed that the content of p-AMPK in liver and muscle in the ST-EtOAc-treated groups was significantly increased compared with the vehicle group. These *in vitro* and *in vivo* results coincided with each other, proving that ST-EtOAc upregulated contents of the p-AMPK and GLUT4, improved glucolipid metabolism, and relieved the IR in KK-Ay so that developing the effects on antidiabetes *in vitro* and *in vivo*.

This study showed ST-EtOAc had promising positive activity on GLUT4 translocation based on a cell-based GLUT4 translocation system. Further investigation *in vitro* and *in vivo* showed that ST-EtOAc indicated a strong stimulation on GLUT4 translocation and enhanced the glucose uptake significantly. *In vitro*, ST-EtOAc improved glucose tolerance, reduced hyperglycemia and reduced insulin levels. ST-EtOAc has the potential to treat diabetes by targeting the AMPK pathway. Furthermore, according to the oral acute toxicity study on ST-EtOAc, the single oral dose LD50 for KM mice was proposed to be more than 6500 mg/kg/day. These results indicate that ST-EtOAc, at the dosages used in our study, did not result in any side effects. Therefore ST-EtOAc can be classified as a non-toxic substance according to the common classification of the relative toxicity of chemicals. In order to identify the main components from ST-EtOAc effectively, 13 compounds had been isolated by the off-line semipreparative HPLC–NMR. Some of these compounds have been reported to be effective bioactive compounds, such as genistin, which has shown an effect on weight gain in pregnant rats (Cao et al., 2015), and quercetin, which has shown to stimulate insulin secretion and reduce the viability of rat INS-1 beta-cells (Kittl et al., 2016). The research suggests that ST-EtOAc has the potential to become an effective agent in the therapy of diabetes mellitus.

## Author Contributions

XY contributed to the conception of the study; MH and SD contributed significantly to analysis and manuscript preparation equally; QH, PZ, QZ, and SZ performed the data analyses and wrote the manuscript equally; XM, CX, and JY helped perform the analysis with constructive discussions equally.

## Conflict of Interest Statement

The authors declare that the research was conducted in the absence of any commercial or financial relationships that could be construed as a potential conflict of interest.
