# Supplementary material for: Hypoglycemic Activity and the Potential Mechanism of the Flavonoid Rich Extract from Sophora tonkinensis Gagnep. in KK-Ay Mice
Source: Front Pharmacol. 2016 Sep 5;7:288. doi: 10.3389/fphar.2016.00288 (PMC5011294; doi:10.3389/fphar.2016.00288)
Supplement: Supplementary file 1 [file Presentation_1.PDF]

Hypoglycemic Activity and the Potential Mechanism of the Flavonoid Rich Extract from *Sophora tonkinensis* Gagnep. in KK-Ay Mice

Mi Huang[a,1], Shihao Deng[a,1], Qianqian Han[a], Ping Zhao[a], Qi Zhou[a], Sijian Zheng[a], Xinhua Ma[a], Chan Xu[a], Jing Yang[a], Xinzhou Yang[a,b,c*]

[a]School of Pharmaceutical Sciences, South-Central University for Nationalities, Wuhan 430074, China

[b]State Key Laboratory of Drug Research, Shanghai Institute of Materia Medica, Chinese Academy of Sciences, 555 Zu-Chong-Zhi Road, Shanghai 201201, China

[c]College of Biological Engineering, Tianjin University of Science and Technology, Tianjin 300457, China

[*]Corresponding authors at: School of Pharmaceutical Sciences, South-Central University for Nationalities, 182 Min-Zu Road, Wuhan 430074, China. Tel.: +86 27 67841196; Fax: +86 27 67841196. E-mail addresses: xzyang@mail.scuec.edu.cn (X.Z. Yang).

[1]These authors contributed to this article equally.

Abstract

This study investigated the active principles, hypoglycemic activity and potential mechanisms of the flavonoid rich extract from *Sophora tonkinensis* Gagnep. (ST-EtOAc) in KK-Ay diabetic mice. An off-line semipreparative LC-NMR and LC-UV-ESIMS protocol was performed to determine 13 flavonoids from ST-EtOAc. ST-EtOAc administrated orally to the KK-Ay mice significantly increased their sensibility to insulin, reduced fasting blood-glucose levels and blood lipid indexes such as triglyceride and cholesterol. Moreover, ST-EtOAc exhibited a strong effect of stimulation on GLUT4 translocation by 2.7 fold in L6 cells. However, the selective AMP-activated protein kinase (AMPK) inhibitor Compound C can completely inhibit the activation of the AMPK pathway and prevent the GLUT4 translocation caused by ST-EtOAc. *In vivo*, phosphorylation of the AMPK expression in the liver and skeletal muscle was measured. The results showed phosphorylation of the AMPK had been improved and GLUT4 expression had been also enhanced. In this paper we conclude that, ST-EtOAc seems to have potential beneficial effects on the treatment of type 2 diabetes mellitus (T2DM) with the probable mechanism of stimulating GLUT4 translocation modulated by the AMPK pathway.

Keywords: Hypoglycemic agents; *Sophora tonkinensis* Gagnep.; KK-Ay mice; GLUT4; p-AMPK

Supporting Information Contents:

S1. Experimental Section

S2. Spectroscopic data of compounds 1-13

S3. The results of related index of KK-Ay mice

S1. Experimental Section

Methodology validation

There have several papers reported that IRAP-mOrange and GLUT4-eGFP could be applied to detect the GLUT4 translocation in L6 (Wang et al., 2009; Zhou et al., 2016; Huang et al., 2016) and 3T3L1 cells (Bai et al., 2007; Jiang et al. 2008). In order to validate the feasibility of our IRAP translocation assay for discovering potential hypoglycemic agents, we have observed the effects when the GLUT4-eGFP or IRAP-marked L6 cells treated with insulin and berberine which are definitely pharmacodynamic GLUT4 agonists. L6 cells which stably express IRAP-mOrange and GLUT4-eGFP were cultured in MEM-α supplemented with 10% fetal bovine serum and 1% antibiotics (100 U/mL penicillin and 100 μg/mL streptomycin) at 37 ^{o}C in 5% CO2. L6 cells was seeded in 48 well plates, and incubated until 100% confluence and then starved in serum-free MEM-α for 2 h. Afterwards, L6 cells were treated with insulin (10 nM) and berberine (5 μM). The cells were taken photos with a laser-scanning confocal microscope LSM 510 (Carl Zeiss, Jena, Germany) to supervise the IRAP-mOrange and GLUT4-eGFP translocation. And the images were captured with 555 nm excitation laser every 10 seconds in first 5 minutes and then every 5 minutes in later 30 minutes.

During the experiment, as time went on, we could observe the green and red fluorescence enhanced significantly after treating with insulin and berberine in L6 cells (Fig. 1). The results showed that GLUT4 and IRAP simultaneously translocated onto the plasma membrane in 30 min when adding the GLUT4 agonist. GLUT4 has mainly been recruited to the PM throughout to the GLUTs storage vesicles (GSV). Three main proteins

stored in GSV are GLUT4, IRAP, and Sortilin (Shi et al., 2005). It was reported that IRAP and GLUT4 displayed a strong colocalization (Kumar et al., 2010; Rubin et al., 2009) in many researches. Thus, detecting the IRAP can indirectly reflect the situation of GLUT4. So our results could be explained that detecting the IRAP-mOrange fluorescence could indirectly reflect the GLUT4 translocation. As the red fluorescence is more conspicuous than green fluorescence for observation, so we choose the IRAP-mOrange fluorescence assay for reflecting GLUT4 translocation.

Western Blot analysis for GLUT4 on plasma membrane

L6 cells (5×10^5 cells) were subcultured into 60 mm dishes and cultured for 7 days to form myotubes in 3 mL of MEM-α with 2% FBS. After incubation, the L6 myotubes were treated with insulin (10 nM), ST-EtOAc, or vehicle (0.1% DMSO) for 12 hours. Plasma membrane fraction of the myotubes was obtained using the method described as previously reported (Nishiumi et al., 2007). Briefly, to prepare the plasma membrane and post-plasma membrane fraction, L6 myotubes were homogenated, and each homogenate was centrifuged at 1,000×g for 10 min at 4 ℃. The precipitate was re-suspended in buffer A containing 1.0 % (v/v) IGEPAL CA-630, placed on ice for 1 h with occasional mixing and was centrifuged at 16,000×g for 20 min at 4 ℃. The supernatant was collected and stored as the plasma membrane (PM) fraction at -80 ℃ until analyses. An equivalent amount of samples were mounted on 10% SDS-polyacrylamide gel electrophoresis (PAGE). The protein transferred electrophoretically to polyvinylidene fluoride membrane (PVDF) (Pall Corporation, Washington, USA) and incubated overnight at 4 ℃ with antibodies specific for GLUT4 and β-Actin. The membranes were washed 3 times (10 min/wash) in TBST.

Immune complexes were incubated with a peroxidase-conjugated antibody for 1 h. The blots were incubated in enhanced chemiluminescence kits (Amersham-Pharmacia, Piscataway, NJ, USA). And the Immunoreactive signals were imaged and quantified with the Gel Image system (Aplegen Inc., Pleasanton, USA). The results were shown in Figure 2.

As shown in Figure. 2, the result showed that after adding ST-EtOAc and insulin, the translocation of GLUT4 on myotube cells membrane had been improved consequently compared with normal control. Corresponding to the level of p-AMPK increasing, we can demonstrate ST-EtOAc stimulate GLUT4 translocation by AMPK signal pathway.

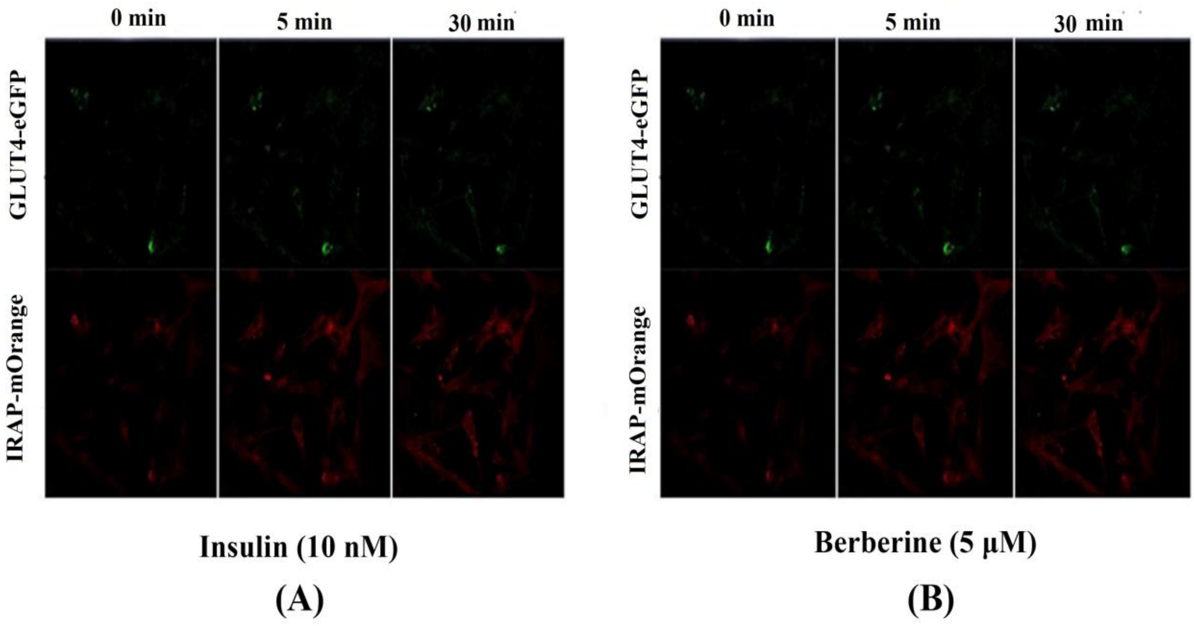

Figure. 1 L6 cells were infected with IRAP-mOrange and GLUT4-eGFP in order to detect externalized GLUT4 translocation by confocal microscopy. (A) Confocal images in L6 cells incubated in the absence (0 min) or presence of insulin for 5min, 30 minutes. (B) Confocal images in L6 cells incubated in the absence (0 min) or presence of berberine for 5 min, 30 minutes.

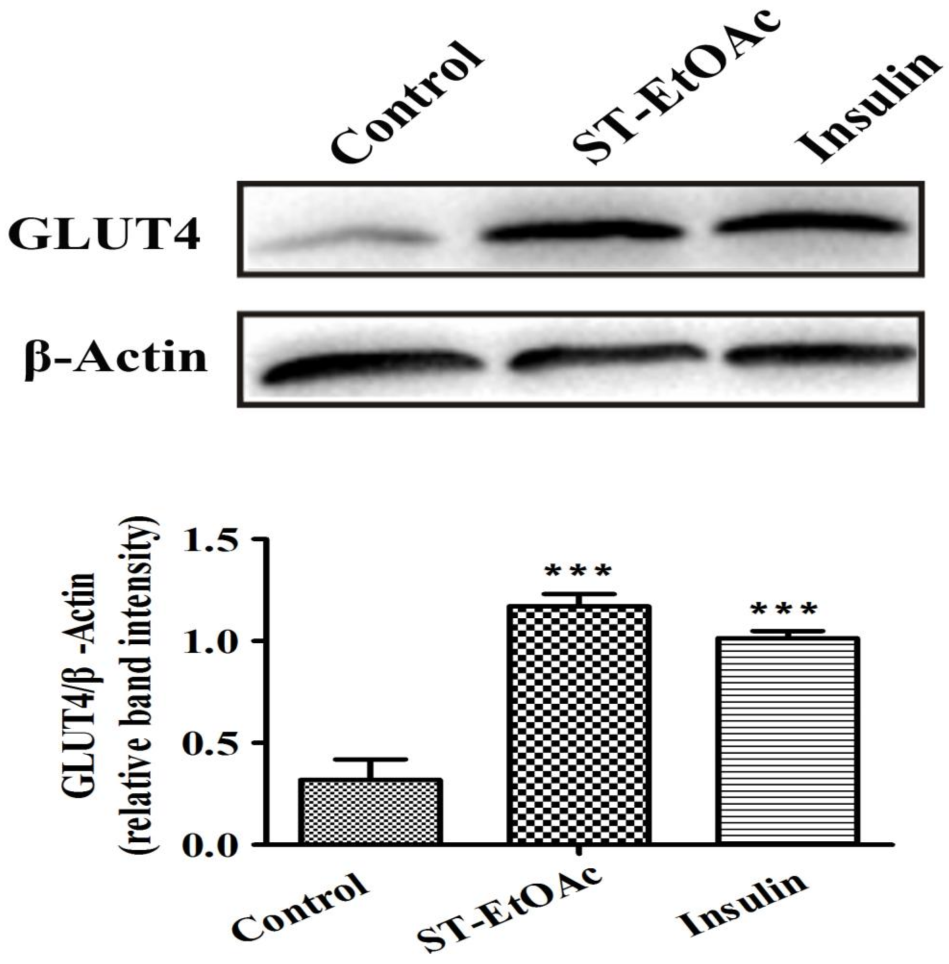

Figure. 2 ST-EtOAc induced an increase of GLUT4 protein level on plasma membrane. ***P≤0.001, compared to normal control.

References

Bai, L., Wang, Y., Fan, J., Chen, Y., Ji, W., Qu, A., Xu, P., James, D. E., Xu, T. (2007). Dissecting multiple steps of GLUT4 trafficking and identifying the sites of insulin action. Cell Metab. 5, 47-57.

Huang, M., Zhao, P., Xiong, M. R., Zhou, Q., Zheng, S. J., Ma , X. H., Xu, C., Yang, J., Yang, X. Z., Zhang, T. C. (2016). Antidiabetic activity of perylenequinonoid-rich extract from Shiraia bambusicola in KK-Ay Mice with Spontaneous Type 2 Diabetes Mellitus. J. Ethnopharmac. 191, 71-81.

Jiang, L., Fan, J., Bai, L., Wang, Y., Chen, Y., Yang, L., Chen, L., Xu, T. (2008). Direct quantification of fusion rate reveals a distal role for AS160 in insulin-stimulated fusion of GLUT4 storage vesicles. J. Biol. Chem. 283, 8508-8516.

Kumar, A., Lawrence, Jr., Jung, D. Y., Ko, H. J., Keller, S. R., Kim, J. K., Magnuson, M. A., Harris, T. E. (2010). Fat cellspecific ablation of rictor in mice impairs insulin-regulated fat cell and whole-body glucose and lipidmetabolism. Diabetes. 59, 1397–1406. doi: 10.2337/db09-1061.

Nishiumi S., Ashida H. (2007). Rapid preparation of a plasma membrane fraction from adipocytes and muscle cells: application to detection of translocated glucose transporter 4 on the plasma membrane. Biosci. Biotechnol. Biochem. 71, 2343-2346.

Rubin, B. R., Bogan, J. S. (2009). Intracellular retention and insulinstimulated mobilization of GLUT4 glucose transporters. Vitam. Horm. 80, 155–192. doi: 10.1016/S0083-6729(08)00607-9.

Shi, J., Kandror, K. V. (2005). Sortilin is essential and sufficient for the formation of glut4 storage vesicles in 3T3-L1 adipocytes. Dev. Cell. 9, 99–108.

Wang, X., Qu, F., Chen, Z., Liang, T., Qu, A. (2009). Labeling and imaging of GLUT4 in live L6 cells with quantum dots. Biochem. Cell. Biol. 87, 687-694.

Zhou, Q., Yang, X. Z., Xiong, M. R., Xu, X. L., Zhen, L., Chen, W. W., Wang, Y., Shen, J. H., Zhao, P., Liu, Q. H. (2016). Chloroquine Increases Glucose Uptake via Enhancing GLUT4 Translocation and Fusion with the Plasma Membrane in L6 Cells. Cell Physiol. Biochem. 38, 2030-2040.

S2. Spectroscopic data of compounds 1-13

Compound 1: Yellow powder; Molecular formula: $C_{15}H_{10}O_{5;}$ ESI-MS (m/z): 270 $[M–H]^{-}$. UV (MeOH) λ_{max}: 259.3nm. ^{1}H-NMR (500 MHz, DMSO-D_6): δ_H 6.21 (1H, d, J = 2.1 Hz, H-6), 6.37 (1H, d, J = 2.1 Hz, H-8), 6.81 (2H, d, J = 8.4 Hz, H-3', H-5), 7.36 (2H, d, J = 8.4 Hz, H-2', H-6'), 8.31(1H, s, H-s), 9.56 (1H, s, H-4'), 10.86 (1H, s, H-7), 12.94 (1H, s, H-5); ^{13}C-NMR (125 MHz, DMSO-D_6): δ_C 180.4 (C-4), 164.4 (C-7), 162.1 (C-5), 157.7 (C-8a), 157.6 (C-4'), 154.1 (C-2), 130.3 (C-2'. C-6'), 122.4 (C-3), 121.4 (C-1'), 115.2 (C-3', C-5'), 104.6 (C-4a), 99.1 (C-6), 93.8 (C-8). By comparison of the MS, UV and NMR spectra with published reference data (Al-Maharik et al., 2008), compound **1** was identified as genistin.

Compound 2: Brown solid; Molecular formula: $C_{22}H_{22}O_{10}$; ESI-MS (m /z): 445 $[M–H]^{-}$; UV (MeOH) λ_{max}: 259nm; ^{1}H-NMR (500 MHz, DMSO-D_6): δ_H 4.27 (2H, dd, J = 6.0, 4.5Hz, H-2), 3.62 (1H, m, H-3), 5.57 (1H, d, J = 7.5 Hz, H-4), 7.36 (1H, d, J = 8.5 Hz, H-5), 6.69 (1H, dd, J = 8.5, 2.5Hz, H-6), 6.55 (1H, d, J = 2.5 Hz, H-8), 6.96 (1H, s, 2'-H), 6.54 (1H, s, 5'-H), 4.83 (1H, d, J = 7.5Hz, H-1''), 5.95 (2H, s, –OCH_2O–); ^{13}C-NMR (125 MHz, DMSO-D_6): δ_C 66.3 (C-2), 40.2 (C-3), 78.1 (C-4), 132.4 (C-5), 110.8 (C-6), 158.5 (C-7), 104.5(C-8), 156.7 (C-9), 114.6 (C-10), 118.7 (C-10), 105.8 (C-2'), 141.6(C-3'), 147.9 (C-4'), 93.7 (C-5'), 154.1 (C-6'), 100.7 (C-1''), 73.6 (C-2''), 76.9 (C-3''), 70.1 (C-4''), 77.5 (C-5''), 61.0 (C-6''), 101.5 (–O-CH_2-O–). By comparison of the MS, UV and NMR spectra with published reference data (Yang et al., 2013), compound **2** was identified as trifolirhizin.

Compound 3: Yellow crystal; Molecular formula: $C_{16}H_{12}O_4$; ESI-MS (m/z): 268 $[M–H]^{-}$; UV (MeOH) λmax: 202, 248, 300 nm; ^{1}H-NMR (500 MHz, DMSO-D_6): δ_H 3.85 (3H, s,

OCH_3), 6.88 (1H, d, J = 2.1 Hz, 8-H), 6.97 (1H, dd, J = 8.8, 2.1 Hz, H-6), 7.01 (2H, d, J = 8.8, 2.1Hz, 3'-H, 5'-H), 7.49 (2H, d, J = 8.8 Hz, 2'-H, 6'-H), 8.08 (1H, J = 8.8 Hz, 5-H), 8.21 (lH, s, 2-H); ^{13}C-NMR (125 MHz, DMSO-D_6): δ_C 154.7 (C-2), 124.3 (C-3), 180.0 (C-4) 127.2 (C-5), 115.1 (C-6), 164.6 (C-7), 102.1 (C-8), 159.6 (C-9), 123.4 (C-l'), 130.0 (C-2'), 113.5 (C-3'), 161.7 (C-4'), 113.5 (C-5'), l30.0 (C-6'), 55.1 (-OCH_3). By comparison of the MS, UV and NMR spectra with published reference data (Yang et al., 2014), compound **3** was identified as ononin.

Compound 4: White amorphous powder; Molecular formula: $C_{24}H_{24}O_{10}$; ESI-MS (m/z): 488 [M–H]$^-$; UV (MeOH) λmax: 259 nm; ^{1}H-NMR (500 MHz, DMSO-D_6): δ_H 4.27 (2H, dd, J = 6.0, 4.5 Hz, H-2), 3.62 (1H, m, H-3), 5.57 (1H, d, J = 7.5 Hz, H-4), 7.36 (1H, d, J = 8.5 Hz, H-5), 6.69 (1H,dd, J = 8.5, 2.5 Hz, H-6), 6.55 (1H, d, J = 2.5 Hz, H-8), 6.96 (1H, s, 2'-H), 6.54 (1H, s, 5'-H), 4.83 (1H, d, J = 7.5 Hz, H-1''), 5.95 (2H, s, –OCH_2O–), 2.02 (3H, s, -OAc); ^{13}C-NMR (125 MHz, DMSO-D_6): δ_C 20.97 (-CH_3), 66.3 (C-2), 40.2 (C-3), 78.1 (C-4), 132.4 (C-5), 110.8 (C-6), 158.5 (C-7), 104.5(C-8), 156.7 (C-9), 114.6 (C-10), 118.7 (C-10), 105.8 (C-2'), 141.6 (C-3'), 147.9 (C-4'), 93.7 (C-5'), 154.1 (C-6'), 100.7 (C-1''), 73.6 (C-2''), 76.9 (C-3''), 70.1 (C-4''), 77.5 (C-5''), 61.0 (C-6''), 101.5 (–O-CH_2-O–), 170.67(C=O).By comparison of the MS, UV and NMR spectra with published reference data (Yang et al., 2014), compound **4** was identified as trifolirhizin 6'-monoacetat.

Compound 5: White powder; Molecular formula: $C_{15}H_{10}O_7$; ESI-MS (m/z): 302 [M–H]$^-$; UV (MeOH) λmax: 206, 285, 354 nm; ^{1}H-NMR (500 MHz, DMSO-D_6): δ_H 12.5 (1H, s, OH-5), 7.67 (1H, d, J = 2.0 Hz, H-2'), 7.54 (1H, dd, J = 2.0, 8.4 Hz, H-6'); ^{13}C-NMR (125 MHz, DMSO-D_6): δ_C 98.2 (C-6), 93.4 (C-8), 103.0 (C-10), 115.1 (C-2'), 115.7 (C-5'), 120.0 (C-6'), 122.0 (C-1'), 135.8 (C-3), 145.1 (C-3'), 146.8 (C-2), 147.8 (C-4'), 156.2 (C-5), 160.8 (C-9), 164.0 (C-7), 175.9 (C-4). By comparison of the MS, UV and NMR spectra

with published reference data (Yi et al., 2002), compound **5** was identified as quercetin.

Compound 6: White crystal; Molecular formula: $C_{16}H_{12}O_5$; ESI-MS (m/z): 284 $[M–H]^-$; UV (MeOH) λmax: 203, 309 nm; ^{1}H-NMR (500 MHz, MeOH-d_4): δ_H 7.36 (1H, d, J = 8.0Hz, H-5), 6.72 (1H, s, H-2'), 6.54 (1H, dd, J = 8.0, 2.0 Hz, H-6), 6.44 (1H, s, H-5'), 6.41 (1H, d, J = 2.4 Hz, H-8), 5.91 (2H, dd, J =11.6, 1.6 Hz, -O-CH_2-O-), 5.47 (1H, d, J = 7.2 Hz, H-4a), 4.22 (1H, dd, J = 10.8, 4.8 Hz, H-3a), 3.64 (1H, t, J = 10.8 Hz, H-2b), 3.48 (1H, m, H-2a); ^{13}C-NMR (125 MHz, MeOH-d_4): δ_C 66.5 (C-2), 40.1 (C-3), 78.5 (C-4), 132.1 (C-5), 109.8 (C-6), 157.0 (C-7), 103.7 (C-8), 156.7 (C-9), 112.7 (C-10), 117.9 (C-1'), 104.7 (C-2'), 141.7 (C-3'), 148.1 (C-4'), 93.8 (C-5'), 156.6 (C-6'), 101.3 (-O-CH_2-O-). By comparison of the MS, UV and NMR spectra with published reference data (Lee et al., 2015), compound **6** was identified as maackiain.

Compound 7: Yellow oil; Molecular formula: $C_{30}H_{36}O_6$; ESI-MS (m/z): 492 $[M–H]^-$; UV (MeOH) λmax: 297, 339 nm; ^{1}H-NMR (500 MHz, DMSO-D_6): δ_H 4.94 (2H, d, J = 12.0 Hz, H-2), 4.50 (1H, d, J = 12.0 Hz, H-3), 7.27 (1H, s, H-2'), 6.68 (1H, d, J = 8.2 Hz, H-5'), 7.28 (1H, dd, J = 8.2 Hz, H-6'); ^{13}C-NMR (125 MHz, DMSO-D_6): δ_C 83.1 (C-2), 72.5 (C-3), 196.5 (C-4), 100.5 (C-4a), 158.8 (C-5), 107.8 (C-6), 163.2 (C-7), 107.0 (C-8), 157.9 (C-8a), 128.8 (C-1'), 129.3 (C-2'), 127.0 (C-3'), 155.1 (C-4'), 115.9 (C-5'), 126.8 (C-6'). By comparison of the MS, UV and NMR spectra with published reference data (Mori-Hongo et al., 2009), compound **7** identified as Lespeflorins B4.

Compound 8: Yellow solid; Molecular formula: $C_{25}H_{24}O_6$; ESI-MS(m/z): 420 $[M–H]^-$; ^{1}H-NMR (500 MHz, DMSO-D_6): δ_H 8.10 (2H, d, J = 8.8 Hz, H-2', H-6'), 7.01 (2H, d, J = 8.8 Hz, H-3', H-5'), 6.72 (1H, d, J = 9.9 Hz, H-4''), 5.62 (1H, d, J = 10.0 Hz, H-5''), 1.45

(1H, s, CH_3-6''), 3.49 (1H, d, J = 7.0 Hz, H-1'''), 5.21 (1H, m, J = 7.0, 6.1Hz, H-2'''), 1.67 (1H, s, CH_3-3'''); ^{13}C-NMR (125 MHz, DMSO-D_6): δ_C 145.4 (C-2), 135.5(C-3), 175.5 (C-4), 153.0 (C-5), 104.9 (C-6), 156.9 (C-7), 107.7 (C-8), 153.6 (C-9), 103.5 (C-10), 123.7 (C-1'), 129.3 (C-2'), 114.1 (C-3'), 161.0 (C-4'), 114.1 (C-5'), 129.3 (C-6'), 115.7 (C-4''), 128.1 (C-5''), 77.8 (C-6''), 28.3 (CH_3-6''), 21.5 (C-1'''), 122.2 (C-2'''), 131.8 (C-3'''), 25.7 (CH_3-3'''), 18.0 (CH_3-3'''). By comparison of the MS, UV and NMR spectra with published reference data (Sutthivaiyakit et al., 2009), compound **8** identified as dehydrolupinifolinol.

Compound 9: Yellow powder; Molecular formula: $C_{25}H_{28}O_4$; ESI-MS (m/z): 392 $[M–H]^-$; UV (MeOH) λmax: 198, 282 nm; ^{1}H-NMR (500 MHz, MeOH-d_4): δ_H 7.78 (1H, d, J = 8.8 Hz, H-5), 7.25 (1H, s, H-2'), 7.26 (1H, d, J = 8.8 Hz, H-6'), 6.88 (1H, d, J = 8.8 Hz, H-5'), 6.58 (1H, d, J = 8.8 Hz, H-6), 5.40 (1H, dd, J = 13.2, 2.8 Hz, H-2), 5.29 (2H, m, H-2'', H-2'''), 3.43 (4H, J = 7.6 Hz, H-1'', H-1'''), 3.03 (1H, dd, J = 16.8, 13.2 Hz, H-3ax), 2.83 (1H, dd, J = 16.8, 2.8 Hz, H-3eq), 1.83 (6H, s, H-4'', H-5''), 1.79 (6H, s, H-4''', H-5'''); ^{13}C-NMR (125 MHz, MeOH-d_4): δ_C 191.9 (C-4), 161.4 (C-7), 161.2 (C-9), 154.4 (C-4'), 135.0 (C-3''), 134.8 (C-3'''), 131.0 (C-1'), 127.8 (C-6'), 127.1 (C-3'), 126.4 (C-5), 125.3 (C-2'), 121.3 (C-2''), 121.0 (C-2'''), 115.7 (C-5'), 114.7 (C-8), 114.6 (C-10), 110.5 (C-6), 79.4 (C-2), 44.0 (C-3), 29.8 (C-1'''), 25.9 (C-4''), 25.8 (C-4'''), 22.3 (C-1''), 18.0 (C-5''), 17.9 (C-5'''). By comparison of the MS, UV and NMR spectra with published reference data (Cho et al., 2012), compound **9** identified as glabrol.

Compound 10: Yellow crystal; Molecular formula: $C_{30}H_{36}O_4$; ESI-MS(m/z): 460 $[M–H]^-$; UV (MeOH) λmax: 313, 283 nm; ^{1}H-NMR (500 MHz, MeOH-d_4): δ_H 5.35 (1H, dd, J = 2.8, 12.8 Hz, H-2), 2.74 (1H, dd, J = 2.8, 16.8 Hz, H-3a), 3.38 (1H, dd, J = 12.8, 16.8 Hz,

H-3b), 7.59 (1H, d, J = 8.4 Hz, H-5), 6.53 (1H, d, J = 8.4 Hz, H-6), 6.54 (1H, d, J = 2.0 Hz, H-2'), 7.07 (1H, d, J = 2.0 Hz, H-6'), 3.31 (2H, d, J = 7.2 Hz, H-1"), 5.18 (1H, t, J = 7.2 Hz, H-2"), 1.63 (6H, s, H-4", -5"), 6.51 (2H, d, J = 7.2 Hz, H-6"), 5.70 (1H, d, J = 7.2 Hz, H-7"), 1.74 (6H, s, H-9", -10"), 1.79 (6H, s, H-14", -15"); ^{13}C-NMR (125 MHz, MeOH-d_4): δ_C 82.1 (C-2), 46.2 (C-3), 193.0 (C-4), 127.8 (C-5), 109.9 (C-6), 162.0 (C-7), 116.0 (C-8), 161.6 (C-9), 114.9 (C-10), 131.4 (C-1'), 125.8 (C-2'), 128.5 (C-3'), 152.8 (C-4'), 128.5 (C-5'), 125.8 (C-6'), 22.4 (C-1"), 122.8 (C-2"), 131.2 (C-3"), 17.6 (C-4"), 25.6 (C-5"), 28.9 (C-6"), 122.9 (C-7"), 132.8 (C-8"), 17.5 (C-9"), 25.6 (C-10"), 28.9 (C-11"), 122.9 (C-12"), 132.8 (C-13"), 17.5 (C-14"), 25.6 (C-15"). By comparison of the MS, UV and NMR spectra with published reference data (Li et al., 2008), compound **10** was identified as sophoranone.

Compound 11: Yellow powder; ESI-MS(m/z): 406 $[M–H]^-$; UV (MeOH) λmax: 224, 295, 360 nm; ^{1}H-NMR (500 MHz, MeOH-d_4): δ_H 1.42, 1.44 (6H, s), 2.77 (1H, dd, J = 17.2, 3.2 Hz, H-3), 3.04 (1H, dd, J = 17.2, 12.8 Hz, H-3), 3.31 (2H, d, J = 7.0 Hz, H-1), 5.20 (1H, m, H-2), 5.30 (1H, dd, J = 12.8, 3.2 Hz, H-2), 5.65 (1H, d, J = 10.0 Hz, H-5), 5.97 (1H, s, H-6), 6.33 (1H, d, J = 10.0 Hz, H-4), 6.80 (1H, d, J = 8.0 Hz, H-5), 7.04 (1H, d, J = 2.0 Hz, H-2), 7.19 (1H, dd, J = 8.0, 2.0 Hz, H-6), 12.32 (1H, s, C_5-OH); ^{13}C-NMR (125 MHz, MeOH-d_4): δ_C 76.5 (C-2), 44.1 (C-3), 194.8 (C-4), 163.1 (C-5), 130.6 (C-6), 158.8 (C-7), 116.9 (C-8), 156.7 (C-9), 132.2 (C-10), 118.4 (C-1'), 123.1 (C-2'), 128.6 (C-3'), 159.6 (C-4'), 118.2 (C-5'), 123.0 (C-6'), 22.8 (C-1"), 103.4 (C-2"), 115.6 (C-3"), 28.7 (C-4"), 28.6 (C-5"), 107.6 (C-1'"), 122.6 (C-2'"), 78.8 (C-3'"), 18.1 (C-4'"). By comparison of the MS, UV and NMR spectra with published reference data (Mizuno et al., 1988), compound **11** was identified as

euchrenone a2.

Compound 12: Brown powder; Molecular formula: $C_{25}H_{26}O_6$; ESI-MS(m/z): 422 $[M–H]^-$; UV (MeOH) λmax: 302, 257 nm; ^{1}H-NMR (500 MHz, MeOH-d_4): δ_H 12.66 (1H, s, OH-5), 10.15 (1H, s, OH-4'), 9.62 (1H, s,OH-7), 9.32 (1H, s, OH-3), 8.00 (2H, d, J = 8.5 Hz, H-2', H-6'), 6.90 (2H, d, J = 8.5 Hz, H-3', H-5'), 5.11 (1H, t, J = 6.5 Hz, H-2'''), 5.14 (1H, t, J = 6.5 Hz, H-2''), 3.50 (2H, d, J = 6.5 Hz, H-1'''), 3.31 (2H, d, J = 6.5 Hz, H-1''), 1.73 (3H, s, CH_3-5'''),1.72 (3H, s, CH_3-5''), 1.61 (6H, s, CH_3-4'', CH_3-4'''); ^{13}C-NMR (125 MHz, MeOH-d_4): δ_C 176.6 (s, C-4), 159.2 (s, C-4'), 158.7 (s, C-7), 155.3 (s, C-5), 151.7 (s, C-9), 146.8 (s, C-2), 135.4 (s, C-3),130.9 (2C, s, C-3'', C-3'''), 129.5 (2C, s, C-2', C-6'), 122.7 (d, C-2'''), 122.3 (d, C-2''), 121.9 (s, C-1'), 115.4 (2C, s, C-3', C-5'), 110.9 (s, C-6), 106.2 (s, C-8), 103.2 (s, C-10), 25.4 (2C, q, C-4'', C-4'''), 21.3 (2C, t, C-1'', C-1'''), 17.9 (2C, q, C-5'', C-5'''). By comparison of the MS, UV and NMR spectra with published reference data (Meragelman et al., 2001), compound **12** was as 6,8-diprenylkaempferol.

Compound 13: Yellow gum; Molecular formula: $C_{30}H_{34}O_4$; ESI-MS(m/z): 460 $[M–H]^-$; UV (MeOH) λmax: 313 nm; ^{1}H-NMR (500 MHz, MeOH-d_4): δ_H 5.39 (1H, dd, J = 2.8, 12.8 Hz, H-2), 2.70 (1H, dd, J = 2.8, 16.8 Hz, H-3a), 3.00 (1H, dd, J = 12.8, 16.8 Hz, H-3b), 7.58 (1H, d, J = 8.4 Hz, H-5), 6.32 (1H, d, J = 8.4 Hz, H-6), 6.91 (1H, d, J = 2.0 Hz, H-2'), 7.05 (1H, d, J = 2.0 Hz, H-6'), 3.31 (2H, d, J = 7.2 Hz, H-1''), 5.18 (1H, t, J = 7.2 Hz, H-2''), 1.63 (6H, s, H-4'', -5''), 6.51 (1H, d, J = 7.2 Hz, H-6''), 5.70 (1H, d, J = 7.2 Hz, H-7''), 1.44 (6H, s, H-9'', -10''), 1.69 (6H, s, H-14'', -15''); ^{13}C-NMR (125 MHz, MeOH-d_4): δ_C 81.1 (C-2), 45.2 (C-3), 191.0 (C-4), 125.2 (C-5), 109.9 (C-6), 162.0 (C-7), 116.0 (C-8), 161.6 (C-9), 114.9 (C-10), 131.4 (C-1'), 125.8 (C-2'), 128.5 (C-3'), 152.8 (C-4'), 128.5 (C-5'),

125.8 (C-6'), 22.4 (C-1''), 122.8 (C-2''), 131.2 (C-3''), 17.6 (C-4''), 25.6 (C-5''), 120.9 (C-6''), 122.9 (C-7''), 131.8 (C-8''), 22.5 (C-9''), 22.6 (C-10''), 28.9 (C-11''), 122.9 (C-12''), 131.8 (C-13''), 16.5 (C-14''), 24.6(C-15''). By comparison of the MS, UV and NMR spectra with published reference data (Li et al., 2008), compound **13** was as sophoranochromene.

Reference

Al-Maharik, N., Botting, N. P. (2008). An efficient method for the glycosylation of isoflavones. Eur. J. Org. Chem. 2008 (33), 5622-5629.

Cho, S., Park, J. H., Pae, A. N., Han, D., Kim, D., Cho, N. C., No, K. T., Yang, H., Yoon, M., Lee, C., Shimizu, M., Baek, N. I. (2012). Hypnotic effects and GABAergic mechanism of licorice (*Glycyrrhiza glabra*) ethanol extract and its major flavonoid constituent glabrol. Bioorg. Med. Chem. 20: 3493-3501.

Lee, J. W., Lee, J. H., Lee, C., Jin, Q. H., Lee, D. H., Kim, Y. S., Hong, J. T., Lee, M. K., Hwang, B. Y. (2015). Inhibitory constituents of *Sophora tonkinensis* on nitric oxide production in RAW 264.7 macrophages. Bioorg. Med. Chem. Lett. 25, 960–962.

Li, X. N., Lu, Z. Q., Chen, G. T., Yan, H. X., Sha, N., Guan, S. H., Yang, M., Hua, H. M., Wu, L. J., Guo, D. A. (2008). NMR spectral assignments of isoprenylated flavanones from *Sophora tonkinensis*. Magn. Reson. Chem. 46, 898-902.

Meragelman, K. M., McKee, T. C., Boyd, M. R. (2001). Anti-HIV prenylated flavonoids from *Monotes africanus*. J. Nat. Prod. 64, 546-548.

Mizuno, M., Tamura, K., Tanaka, T., Iinuma, M. (1988). Three prenylflavanones from *Euchresta japonica*. Phytochemistry. 27, 1831-1834.

Mori-Hongo, M., Takimoto, H., Katagiri, T., Kimura, M., Ikeda, Y., Miyase, T. (2009). Melanin synthesis inhibitors from *Lespedeza floribunda*. J. Nat. Prod. 72, 194-203.

Sutthivaiyakit, S., Thongnak, O., Lhinhatrakool, T., Yodchun, O., Srimark, R., Dowtaisong, P., Chuankamnerdkarn, M. (2009). Cytotoxic and antimycobacterial prenylated flavonoids from the roots of *Eriosema chinense*. J. Nat. Prod. 72, 1092-1096.

Yang, X. Z., Yang, J., Wang, C., Wan, J. F., Yuan, J. Q., Ren, Y. S. (2014). Sodium - dependent glucose cotransporter 2 (SGLT2) inhibitors from *Sophora flavescens*. J. Yunnan. Univ. (Natural Sciences). 36, 267–272.

Yi, J. H., Zhang, G. L., Li, B. G. (2002). Studies on the chemical constituents of *Pseudotsuga sinensis*. Acta. Pharm. Sin. 37, 352-354.

S3. The results of related index of KK-Ay mice

Table. 1 The effects of ST-EtOAc on body weight. +++P≤0.001 compared to normal control; ***P≤0.001, **P≤0.01, *P≤0.05 compared to T2DM mice treated with vehicle.

	Body weights (g)					
Time (weeks)	Normal control	Vehicle control	Metformin (200 mg/Kg)	ST-EtOAc (60 mg/Kg)	ST-EtOAc (120 mg/Kg)	ST-EtOAc (240 mg/Kg)
0	28.2 ±2.5	42.9 ±4.7^{+++}	42.4 ±4.1	43.7 ±5.1	42.6 ±4.3	43.5 ±3.9
1	27.7 ±3.2	43.7 ±5.1^{+++}	40.6 ±3.7	40.8 ±3.3	40 ± 2.6*	38.3 ±3.1**
2	27.8 ±2.9	44.3 ±3.9^{+++}	38.8 ±3.2**	38.9 ±2.9*	37 ± 2.4**	36.2 ±2.3***
3	28.4 ±3.4	42.6 ±2.7^{+++}	37.9 ±2.8**	38.1 ±3.1**	36.2 ± 2.8***	34.6 ±2.9***
4	27.9 ±2.8	42.4 ±2.8^{+++}	36.2 ±2.3**	36.3 ±2.4**	33.4 ± 1.9***	33.4 ±1.8***

Table. 2 The effects of ST-EtOAc on food intake.

	Food intake (g/d/mouse)					
Time (weeks)	Normal control	Vehicle control	Metformin (200 mg/Kg)	ST-EtOAc (60 mg/Kg)	ST-EtOAc (120 mg/Kg)	ST-EtOAc (240 mg/Kg)
0	3.9 ±0.8	7.4 ±1.9	8.1 ±1.7	7.6 ±1.6	7.4 ±1.7	7.1 ±1.2
1	4.1 ± 1.1	7.9 ±2.2	7.6 ± 1.8	7.5 ± 2.1	7.8 ± 2.2	7.6 ± 2.3
2	5.2 ± 1.5	7.9 ± 2.1	7.4 ± 2.0	7.6 ± 0.9	7.5 ± 1.9	7.5 ± 1.8
3	4.6 ± 1.8	7.6 ± 2.5	7.5 ± 1.6	7.7 ± 1.6	7.6 ± 0.9	7.2 ± 1.7
4	4.7 ±1.4	7.7 ± 1.8	7.4 ± 2.1	7.2 ± 1.9	7.5 ± 1.8	7.2 ± 2.2

Table. 3 The effects of ST-EtOAc on fasted blood glucose level. +++P≤0.001 compared to normal control; ***P≤0.001, **P≤0.01, *P≤0.05 compared to T2DM mice treated with vehicle.

	Blood glucose levels (mmol/L)					
Time (weeks)	Normal control	Vehicle control	Metformin (200 mg/Kg)	ST-EtOAc (60 mg/Kg)	ST-EtOAc (120 mg/Kg)	ST-EtOAc (240 mg/Kg)
0	6.3 ±1.8	17.3 ± 2.4^{+++}	17.6 ± 2.8^{+++}	17.5 ± 2.5^{+++}	18.4 ±2.6^{+++}	17.6 ±3.1^{+++}
1	6.2 ± 1.4	18.2 ± 3.1^{+++}	15.7 ± 2.4*	16.5 ± 1.8*	15.5 ± 1.9**	15.2 ± 2.1**
2	6.2 ± 2.0	18.3 ± 2.8^{+++}	13.8 ± 1.9**	14.8 ± 2.3**	13.6 ± 1.7***	13.1 ± 2.9***
3	6.0 ± 1.5	18.3 ± 2.5^{+++}	11.9 ± 2.2***	12.7 ± 1.9***	11.3 ± 1.5***	10.6 ± 2.0***
4	5.9 ± 0.9	18.7 ± 2.9^{+++}	9.6 ± 1.9***	10.1 ± 1.4***	9.8 ± 1.2***	8.6 ± 1.9***

Table. 4 The effects of ST-EtOAc on OGTT. +++P≤0.001 compared to normal control; ***P≤0.001, **P≤0.01, *P≤0.05 compared to T2DM mice treated with vehicle.

	Blood glucose levels (mmol/L)					
Time (min)	Normal control	Vehicle control	Metformin (200 mg/Kg)	ST-EtOAc (60 mg/Kg)	ST-EtOAc (120 mg/Kg)	ST-EtOAc (240 mg/Kg)
0	6.3 ± 1.3	18.2 ± 2.5+++	11.9 ± 2.1***	12.7 ± 2.9**	11.3 ± 2.1***	10.6 ± 1.9***
30	10.1 ± 1.9	27.1 ± 3.7+++	20.8 ± 2.9*	21.1 ± 3.2*	19.5 ± 2.9**	17.8 ± 2.2**
60	7.9 ± 1.2	25.3 ± 3.9+++	15.1 ± 2.7***	15.3 ± 2.1***	14.8 ± 2.2***	12.6 ± 2.7***
120	6.6 ± 1.8	24.5 ± 2.2+++	12.7 ± 1.9***	12.7 ± 1.8***	13.2 ± 1.8***	9.8 ± 0.9***

Table. 5 Effects of ST-EtOAc on TC, TG, HDL-C, LDL-C and insulin levels in the serum. +++P≤0.001, ++P≤0.01 compared to normal control; ***P≤0.001, **P≤0.01, *P≤0.05 compared to T2DM mice treated with vehicle.

	Normal control	Vehicle control	Metformin (200 mg/Kg)	ST-EtOAc (60 mg/Kg)	ST-EtOAc (120 mg/Kg)	ST-EtOAc (240 mg/Kg)
TC (mg/dL)	75.3 ±8.9	152.3 ±26.8+++	104.7 ±28.8***	131.7 ±43.2	124.5 ±33.8**	107.3 ±21.8***
TG (mg/dL)	97.7 ±10.2	177 ±43.2+++	114.8 ±21.1***	176.2 ±35.8	146.8 ±36.2*	124.7 ±32.7***
FFA (mg/dL)	55.7 ±6.8	119 ±10.8++	62.4 ±9.8**	91.4 ±8.0	78.1 ±9.9*	69.5 ±6.0**
HDLC (mg/dL)	203.7 ±56.2	92.7 ±8.7+++	152.3 ±34.6***	111.3 ±10.2	126.3 ±26.8	142.3 ±21.4***
LDLC (mg/dL)	75 ±10.3	138 ±17.9+++	111.1 ±11.3**	122.3 ±11.4**	115.9 ±12.3**	98.3 ±9.8**
Insulin (mU/L)	38.7 ±5.9	50.2 ±4.9+++	39.3 ±5.4***	46.7 ±3.9**	43.1 ±5.2**	40.1 ±3.2***

Table. 6 TC, TG and FFA levels in mice liver and muscle tissue. +++P≤0.001, ++P≤0.01 compared to normal control; ***P≤0.001, **P≤0.01, *P≤0.05 compared to T2DM mice treated with vehicle.

	Liver			Muscle		
	TC (mg/dL)	TG (mg/dL)	FFA (mg/dL)	TC (mg/dL)	TG (mg/dL)	FFA (mg/dL)
Normal control	61.3 ±7.8	84.6 ±8.3	20.4 ±5.2	21.9 ±4.1	28.1 ±6.3	9.7 ±2.3
Vehicle control	97.4 ±8.8+++	168.75 ±17.2+++	56.3 ±4.3+++	38.8 ±7.1+++	42.1 ±6.7+++	22.9 ±4.1++
Metformin (200 mg/Kg)	74.2 ±10.1**	89.7 ±9.2***	29.6 ±7.8***	22.4 ±6.2***	29.4 ±4.2***	13.6 ±4.4*
ST-EtOAc (60 mg/Kg)	82.7 ±8.5*	109.4 ±10.9*	47.3 ±8.2	28.7 ±5.8*	40.4 ±7.1	20.1 ±2.8
ST-EtOAc (120 mg/Kg)	72.9 ±9.7**	94.5 ±7.2**	39.5 ±5.9*	25.3 ±4.2**	31.9 ±5.2**	16.0 ±5.1
ST-EtOAc (240 mg/Kg)	67.5 ±5.9***	82.1 ±7.7***	33.85 ±6.1**	21.4 ±5.9**	25.7 ±3.8***	13.9 ±5.2*
